# A staged zebrafish–mouse screening strategy for antiseizure compound prioritization

**DOI:** 10.1016/j.neuropharm.2026.111085

**Published:** 2026-06-25

**Authors:** Charleston F. Christie, Emmaline Bendell, Ludivine Renaud, Tucker Williamson, Xiaoyang Li, Richard A. Himes, Lyndsey C. Prosser, Sharon F. Edwards, Cameron S. Metcalf, Peter J. West, Karen S. Wilcox, C. James Chou, Sherine S.L. Chan

**Affiliations:** a Neuroene Therapeutics, Kensington, MD, 20895, USA; b Medical University of South Carolina, Charleston, SC, 29425, USA; c Anticonvulsant Drug Development (ADD) Program, Department of Pharmacology & Toxicology, University of Utah, 84112, USA

## Abstract

Epilepsy affects more than 50 million people worldwide, and the identification of new antiseizure medications (ASMs) with adequate blood-brain barrier (BBB) penetration remains a major challenge. The pentylenetetrazol (PTZ)-induced zebrafish model is widely used for early ASM screening, but most studies use larvae at 7 days post-fertilization (dpf) or earlier, before later stages of BBB maturation are fully assessed. Here, we evaluated whether staged testing in 7-, 14-, and 21-dpf zebrafish could improve prioritization of CNS-active compounds before mammalian testing. Thirty-one Vitamin K (VK) analogs were screened in 7-dpf zebrafish using PTZ-induced seizure-like hyperlocomotor activity as a phenotypic endpoint. Seventeen compounds significantly reduced PTZ-induced hyperlocomotor activity at 7 dpf. Based on quantitative selection criteria, three compounds were advanced to dose-response studies in older zebrafish, and two compounds retained activity at 14 and 21 dpf. These compounds were then evaluated in the mouse 6 Hz seizure model. Both compounds showed protection at 32 mA, whereas only compound 3d remained active in the higher-intensity 44 mA model. Brain-to-plasma analysis confirmed CNS exposure for 3d and 3l, with ratios of 2.64 and 0.853, respectively. These findings support later-stage zebrafish screening as a practical phenotypic prioritization step for selecting CNS-active compounds for mammalian seizure models, while highlighting the need for direct mammalian validation and further PK/PD characterization.

## 1. Introduction

As the fourth most prevalent neurological condition, epilepsy affects 50 million people worldwide. However, around a third have drug resistance with the FDA-approved antiseizure medications (ASMs). Epileptic patients who take ASMs often struggle with side effects such as fatigue, depression, and weight gain (Stroke, 2019). With the prevailing disorder, expensive medical care, and uncertainty of effective pharmaceuticals, novel ASMs are continually being developed to target epilepsy. For 5000–10,000 new compounds that are manufactured in the laboratory, only one drug will reach the market over 7 to 15 years (Bryda, 2013; Cassar et al., 2020; Van Norman, 2016). With epilepsy, the additional hurdles for scientists are to create drugs that can penetrate the blood-brain barrier (BBB) and use the most efficient animal model to demonstrate this permeation quickly and effectively (Bowman and Zon, 2010). Less than 2% of approved small-molecule drugs effectively penetrate the BBB (Pardridge, 2005).

The BBB is a highly regulated interface in the central nervous system that controls the movement of molecules between the blood and the brain. As a safety net, it prevents toxic elements from accessing the brain (Jeong et al., 2008; Zeng et al., 2017). It is formed by brain microvascular endothelial cells connected by tight junctions, together with supporting pericytes, astrocytic endfeet, and basement membrane components. These tight junctions restrict paracellular diffusion and limit the entry of many circulating molecules and drugs into the brain (Daneman and Prat, 2015; Fleming et al., 2013). BBB development and function vary across species and developmental stages. In both zebrafish and mice, BBB endothelial cells form tight junctions and share conserved barrier features with the human BBB, supporting their use as model systems for studying CNS drug penetration and seizure-related pharmacology (Grone and Baraban, 2015; Gu and Dalton, 2017; O'Brown et al., 2018). Zebrafish have become a highly utilized vertebrate animal model to determine the effects of potential ASMs, as the zebrafish recapitulates what is seen in mammalian seizures (Baraban et al., 2005, 2007; Burrows et al., 2020; Griffin et al., 2016; Grone and Baraban, 2015; Hortopan et al., 2010). Zebrafish breeders can produce clutches up to 300 embryos per week, and since embryos and larvae are small (2–7 mm in size) they can easily fit in petri dishes and multi-well plates (Kimmel et al., 1995), allowing for high-throughput compound screening. The BBB in zebrafish appears to form between 3 and 10 days post-fertilization (dpf). Using fluorescent tracers to study BBB leakage, O'Brown et al. found that the BBB is impenetrable by day 5 in zebrafish (O'Brown et al., 2019). Other studies observed that tight junctions, astrocytes, and pericytes appeared fully developed at 10-dpf, yet were absent at younger ages (Fleming et al., 2013), making this a predominantly recognized stage of full BBB development. 3–7-dpf zebrafish are frequently used in screens, yet there is no clear age group that best utilizes novel ASMs adapted for mammals.

Many laboratories use zebrafish to model seizures and screen for potential ASMs (Afrikanova et al., 2013; Banote et al., 2013; Baraban et al., 2007; Cassar et al., 2020; Hortopan et al., 2010; Ibhazehiebo et al., 2018; Kundap et al., 2017; Rahn et al., 2014), and recent FDA-approved ASMs have shown efficacy in zebrafish models (Chiron, 2019; Li et al., 2021; Thornton et al., 2020). Limited novel compounds with successful outcomes in zebrafish and rodents include Magnolol, a plant extract used in traditional Chinese medicine, and Vitamin K (VK) analogs (Li et al., 2020a, 2020b; Rahn et al., 2014). Magnolol and VK analogs have been screened in 5- and 7-dpf zebrafish and tested in parallel with 6 Hz mouse seizure models, resulting in protection against convulsions in both animal species. We hypothesized that positive hits from this earlier age range in zebrafish may continue to have efficacy issues in mice unless further tests are conducted in older fish with a fully matured BBB. In this study, we tested novel VK analogs for the reduction of PTZ-induced seizure-like hyperlocomotor activity across three zebrafish age groups and compared these findings with comparable studies in adult mice to aid our understanding of the drugs’ translatability to mammalian models.

## 2. Methods and materials

### 2.1. Zebrafish

Wild-type zebrafish (*Danio rerio* AB strain) were obtained from the Zebrafish International Resource Center (supported by P40 RR012546 from NIH-NCRR). Zebrafish were maintained and crossed according to methods in “A guide for the laboratory use of zebrafish” (Westerfield, 2000). Fertilized eggs were collected and placed in E3 embryo medium and kept in an incubator set at 28.5°C with a 14/10 h light/dark cycle (Kimmel et al., 1995). After 72-hpf, larvae were transferred to beakers containing habitat water. All zebrafish studies were approved by the Institutional Animal Care and Use Committee (protocol #2018–00278) and performed in accordance with the guidelines.

### 2.2. Induction and monitoring of hyperlocomotor activity in zebrafish

Experimental comparisons were from 7-, 14-, and 21-dpf fish within the same clutch. PTZ-induced seizure-like hyperlocomotor activity in zebrafish was quantified by measuring total distance traveled (Baraban et al., 2005). This procedure has been conducted in previous studies (Abbott et al., 2002; Li et al., 2020b; Rahn et al., 2014). Larvae from the same clutch were distributed across treatment groups and plate positions to reduce positional bias. Automated video tracking was used for locomotor quantification. Full blinding was not performed and is acknowledged as a limitation. All compounds dissolved in DMSO and further diluted in E3 buffer (5 mM NaCl, 0.17 mM KCl, 0.33 mM CaCl_2_, and 0.33 mM MgSO_4_). The final concentration of DMSO was <0.5%. The larvae were incubated at 28.5°C for 1 h in VK analogs with a dose range of 1.25 μM to 20 μM. After the incubation period, PTZ was added to yield a final concentration of 10–15 mM in a total volume of 500 μL per well (10 mM PTZ for 7-dpf zebrafish; 15 mM PTZ for 14- and 21-dpf zebrafish) to induce seizure-like hyperlocomotor activity. The plate was immediately transferred to the Daniovision instrument (Noldus) to track the total distance traveled by zebrafish over 15 min. At termination, zebrafish were visually monitored for startle response and survival while gently tapping the multi-well plate. Ethovision XT software version 14 (Noldus) was used to record live video and measure locomotor activity. The total movement in millimeters was measured in 1-min time bins and compared with PTZ control groups.

**Fig. 1.**
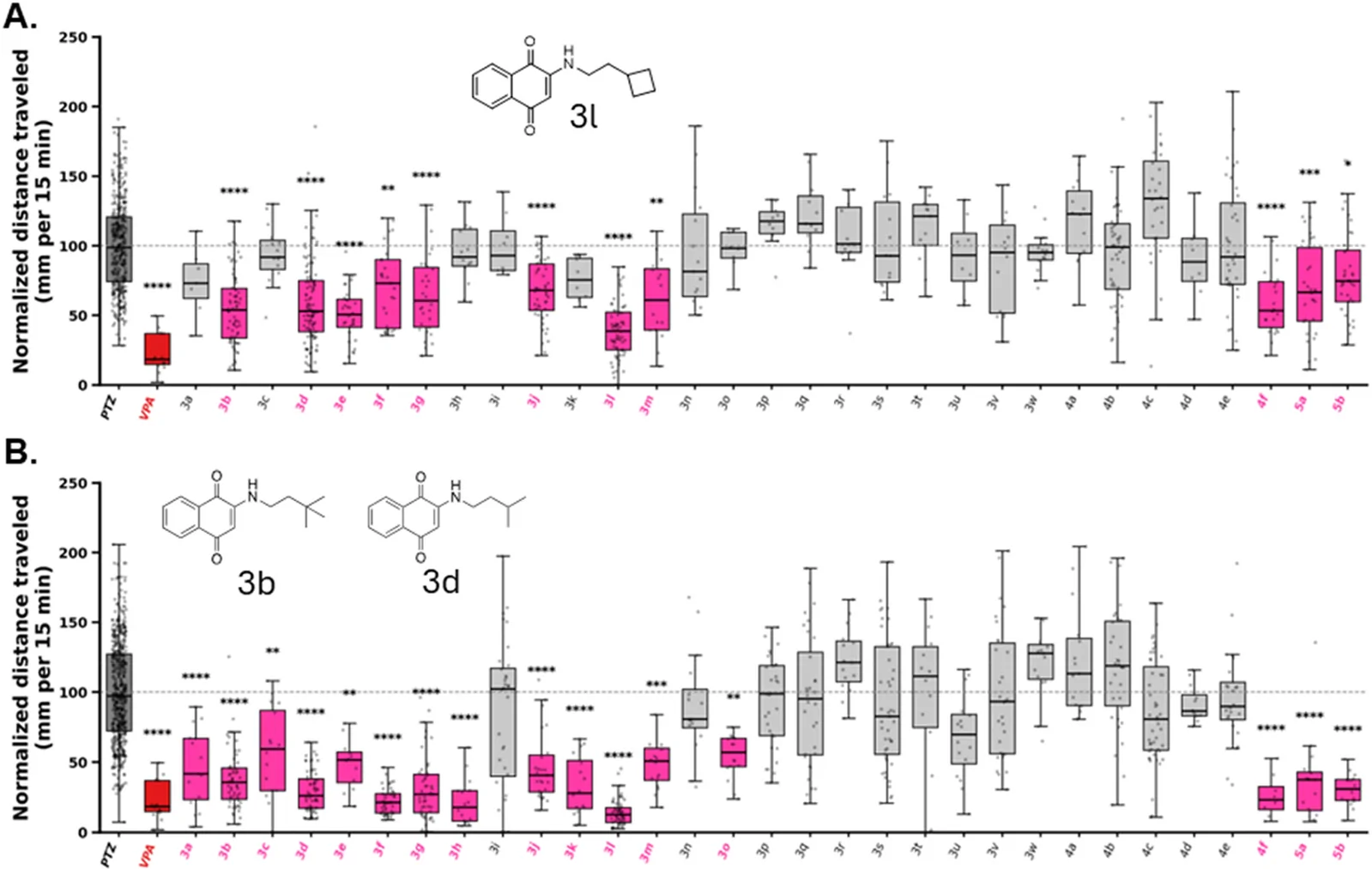
7 dpf zebrafish were pretreated with thirty-one compounds for 1 h before the addition of PTZ, and movements were traced for a course of 15 min. (A and B.) All compound concentrations were in the micromolar range: 5 (A) and 10 (B), except for VPA at 10 mM. The graphs show the distance moved per well by zebrafish when treated with the VK analogs. Total distance traveled was measured in millimeters and normalized to the PTZ control group. *p < 0.05, **p < 0.01, ***p < 0.001, ****p < 0.0001 versus PTZ. Data are shown as box-whisker plots with all individual values. Boxes indicate the interquartile range, center lines indicate the median, and whiskers indicate 1.5 × IQR. Statistical significance was determined using the Kruskal-Wallis test followed by Dunn's multiple comparisons test versus PTZ control with Holm correction. n = 48–124.

**Fig. 2.**
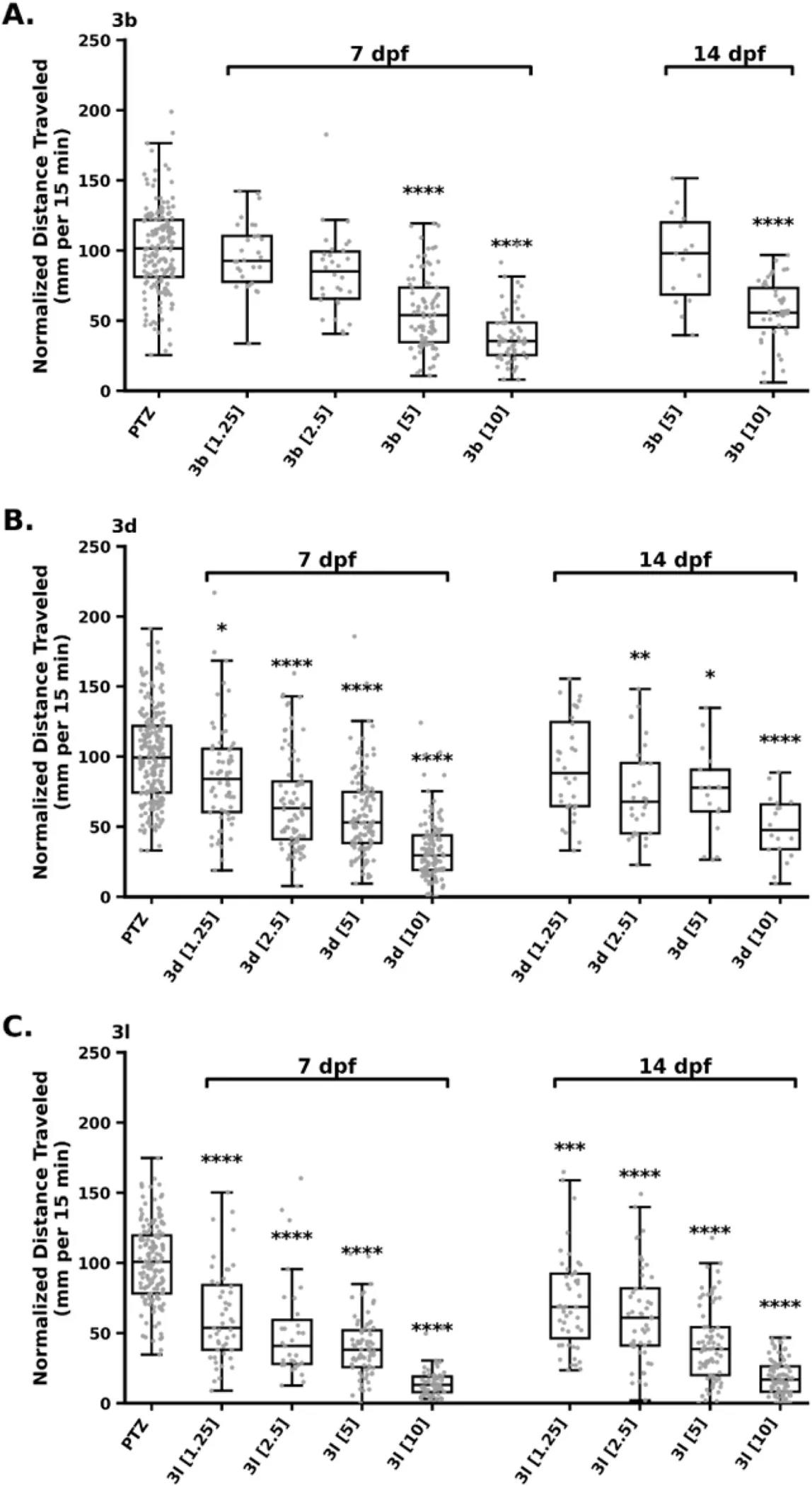
Locomotor activity averages in PTZ-induced zebrafish. (A-C.) 7-dpf and 14-dpf zebrafish were pretreated with compounds (3b, 3d, and 3l) for 1 h prior to the addition of PTZ, and movements were traced for a course of 15 min. (A.) 3b decreased hyperlocomotor activity at 2.5, 5, and 10 μM in 7 dpf and 10 μM in 14 dpf zebrafish. (B and C.) 3d and 3l significantly reduce PTZ-induced hyperlocomotor activity in a dose-dependent manner on 7- and 14-dpf zebrafish. The graphs show the distance moved per well by zebrafish when treated with the VK analogs. Total distance traveled was measured in millimeters and normalized to the PTZ control group. All VK analog concentrations were in micromolar. Statistical significance was determined using the Kruskal-Wallis test followed by Dunn's multiple comparisons test versus PTZ control with Holm correction. Data are shown as box-whisker plots with all individual values. Boxes indicate the interquartile range, center lines indicate the median, and whiskers indicate 1.5 × IQR. *p < 0.05, **p < 0.01, ***p < 0.001, ****p < 0.0001, n = 48–124.

**Fig. 3.**
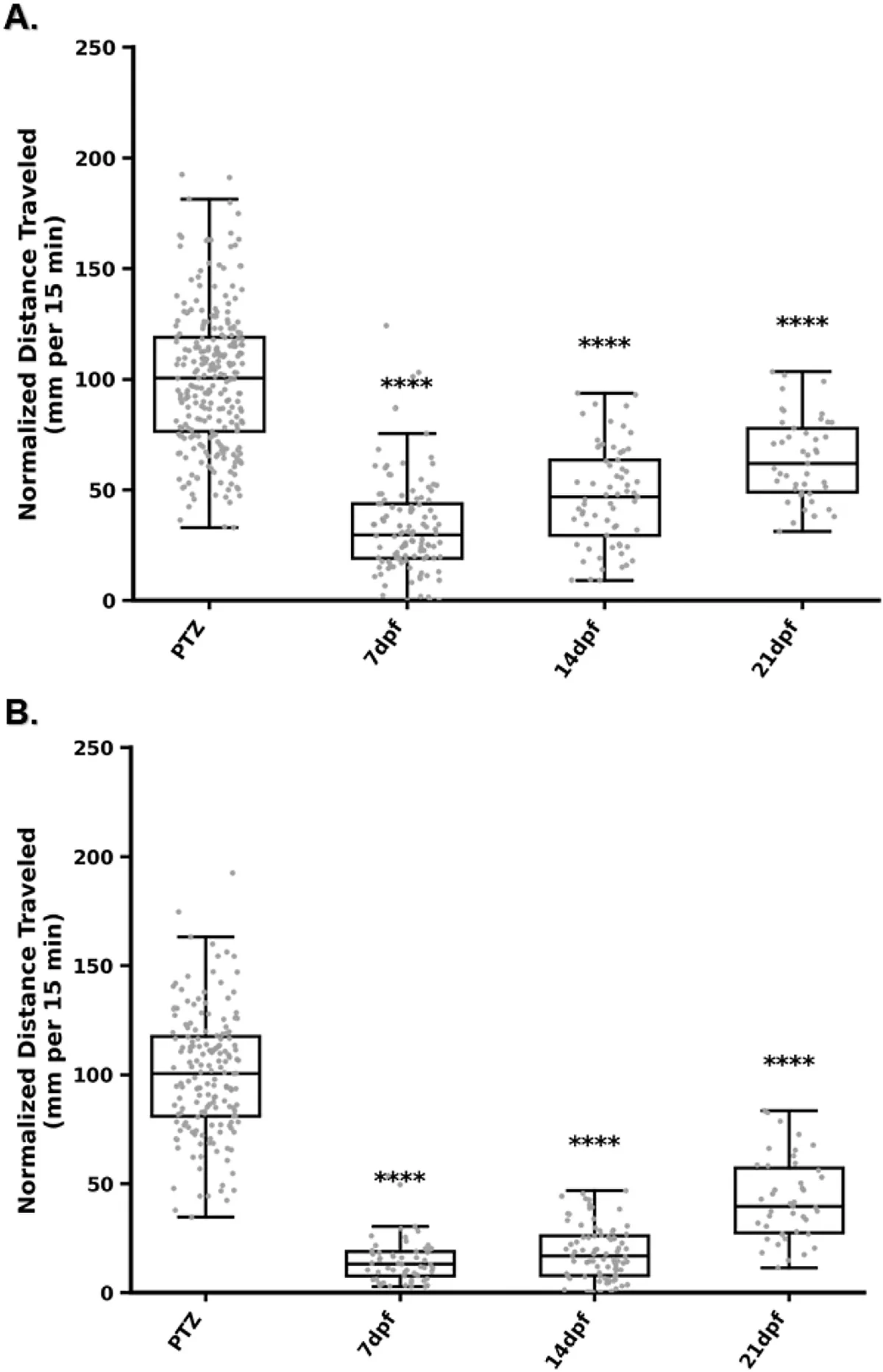
Comparative graphs of PTZ-treated zebrafish using 3d and 3l. Fish were treated with the compound for 1 h prior to the addition of PTZ. (A.) 10 μM 3d tested on zebrafish at 7-, 14-, and 21-dpf. (B.) 10 μM 3l tested on zebrafish at 7-, 14-, and 21-dpf. Total distance traveled was measured in millimeters and normalized to the PTZ control group. Locomotor activity was normalized to 100%, and statistical significance was determined using the Kruskal-Wallis test followed by Dunn's multiple comparisons test versus PTZ control with Holm correction. Data are shown as box-whisker plots with all individual values. Boxes indicate the interquartile range, center lines indicate the median, and whiskers indicate 1.5 × IQR. ****p < 0.0001, n = 48–124. VK-treated fish data were pooled and were normalized to the PTZ control group baseline values at 100%.

Statistical analysis for zebrafish locomotor assays was performed using non-parametric methods because the data were not assumed to be normally distributed. For Figs. 1–3, differences among groups were first evaluated using the Kruskal-Wallis test. When the overall test indicated group differences, post hoc comparisons were performed using Dunn's multiple comparisons test, with each treatment group compared only with the PTZ control group. Holm correction was applied to adjust for multiple comparisons. Data are shown as box-whisker plots with all individual values. Boxes represent the interquartile range (Q1–Q3), the center line represents the median, and whiskers indicate 1.5 × IQR. Statistical significance versus PTZ is indicated as *p < 0.05, **p < 0.01, ***p < 0.001, and ****p < 0.0001 (GraphPad Prism). For initial compound screens, compounds were prioritized based on three criteria: 1) statistically significant reduction in PTZ-induced seizure-like hyperlocomotor activity versus PTZ control after non-parametric analysis, 2) magnitude of median reduction in normalized distance traveled, and 3) consistency of activity across the 5 and 10 μM screening concentrations.

### 2.3. 6 Hz mouse electroshock seizure model

The Anticonvulsant Drug Development (ADD) Program at the University of Utah conducted the 6 Hz mouse model in accordance with NIH procedures. CF-1 mice are maintained in individual cages on a 12-h light/12-h dark cycle at 22±°C and 50 ± 20% humidity. Water was available as needed, and animals were fed a standard laboratory rodent diet. The 6 Hz mouse model is regularly used to test novel ASMs (Metcalf et al., 2017). An acute form of electroshock stimulus at 6 Hz for 3 s is applied to 8 mice per condition. The test uses currents of 32 or 44 mA. At 44 mA, few clinically used ASMs are protective, compared to 32 mA, where several ASMs conferred protection (Barton et al., 2001). Mice were considered protected as a binary endpoint if they did not display the characteristic seizure behaviors after stimulation, including involuntary movements of the jaw, forelimbs, vibrissae, and tail. The number of protected animals was normalized to the total number of animals tested per dose group. Seizures were induced at 25 min after intraperitoneal (i.p.) drug administration, ranging from 100 to 600 mg/kg in 5% DMSO: 95% miglyol solution. The number of rodents protected against seizures were compared with the total number of animals used in the experiment. Probit analysis was conducted using mean ± standard error of the mean to measure the median effective dose (ED50), 95% confidence interval, and the slope of the regression line.

### 2.4. Pharmacokinetics bioanalysis

I.V. injections were conducted via the tail vein of CF-1 mice, with doses of 2–5 mg/kg 3d and 3l dose formulations were 20% DMA:40% PEG300:40% H_2_O. 80 μL or less of blood was collected via the saphenous or jugular vein in Greiner MiniCollect K_2_EDTA vials and kept on ice for 30 min. Samples were collected at 30 min and precipitated with a 3:1 mixture of acetonitrile to plasma. Tubes were centrifuged for 10 min at 3000 × g and stored at −70°C. Tissue samples were homogenized in 3:1 solution of 7.4 pH PBS buffer. An additional 3:1 solution of acetonitrile and internal standard was added to each tissue sample. The vials were vortexed and centrifuged for 10 min at 3000 × g. Standards and quality-control samples were prepared from a 1 mg/mL methanol stock solution and subsequently diluted to 1 ng/mL to 10 μg/mL. All supernatants from plasma and tissue samples were analyzed by Liquid Chromatography – Tandem Mass spectrometry. Plasma and brain concentrations were measured at 30 min post-dose, and brain-to-plasma ratios were calculated. Data were represented by individual animals’ mean, standard deviation, and coefficient of variation.

## 3. Results

New and previously established VK analogs were tested on three different age groups in zebrafish. First, 7-dpf zebrafish larvae were submerged in thirty-one selected VK compounds (Supplementary Table 1 and Fig. 1) in two concentrations (5 and 10 μM) 1 h prior to pentylenetetrazol (PTZ)-induced seizure-like hyperlocomotor activity. The PTZ-only control fish had greatly increased locomotor movement, circling the wells within 5 min. The total distance traveled was compared between compound-treated and control fish treated with PTZ only. Out of the thirty-one compounds, 17 compounds provided significant reduction (p < 0.05) against PTZ-induced hyperlocomotor activity in 7-dpf larvae. The 14-dpf zebrafish were later treated with the 17 effective VK analogs at 2 concentrations (5 and 10 μM). Quantitative prioritization was based on median reduction in PTZ-induced locomotor activity at both 5 and 10 μM and statistical significance versus PTZ after Kruskal-Wallis analysis followed by Dunn's multiple comparisons with Holm correction. Compounds 3l, 3d, and 3b showed the most consistent activity across both concentrations. The median normalized distance traveled for 3l was 38.8% at 5 μM and 12.7% at 10 μM, with adjusted p values of 4.76 × 10^−34^ and 7.68 × 10^−45^. For 3d, the medians were 53.0% and 26.1%, with adjusted p values of 4.70 × 10^−25^ and 1.36 × 10^−31^. For 3b, the medians were 53.8% and 35.8%, with adjusted p values of 1.42 × 10^−20^ and 3.87 × 10^−23^. These three compounds were therefore selected for dose-response testing in older zebrafish. The three compounds were further examined for dose response using concentrations 1.25, 2.5, 5, and 10 μM in the 7-dpf group. The 7-dpf fish had a dose-dependent response, with all three compounds reducing seizure-like hyperlocomotor activity by 5–75% when compared to PTZ-only controls (Fig. 2). For 14-dpf larvae, only two analogs (3d and 3l) reduced hyperlocomotor activity at both 5 and 10 μM (Fig. 2). Furthermore, 3d and 3l also significantly reduced hyperlocomotor activity in 14-dpf zebrafish at 1.25 and 2.5 μM (Fig. 2). 3d and 3l were proportional across doses in the 14-dpf fish; however, 3l had the strongest effect at 10 μM, resulting in an 80% decrease in hyperlocomotor activity. The two most effective compounds, 3d and 3l, were further tested in 21-dpf zebrafish. Both VK analogs at 10 μM reduced PTZ-induced hyperlocomotor activity in the older larvae. At 10 μM, 3l conferred a greater reduction in seizure-like hyperlocomotor activity than 3d at 7-, 14-, and 21-dpf (Fig. 3). 3d showed a gradual decrease in efficacy, with travel distances rising by 10–15% across age groups. 3l′s travel distances remained low at 15% at 7 and 14-dpf, which increased by 30% at 21-dpf.

### 3.1. Protection in the 6 Hz mouse seizure model

**Fig. 4.**
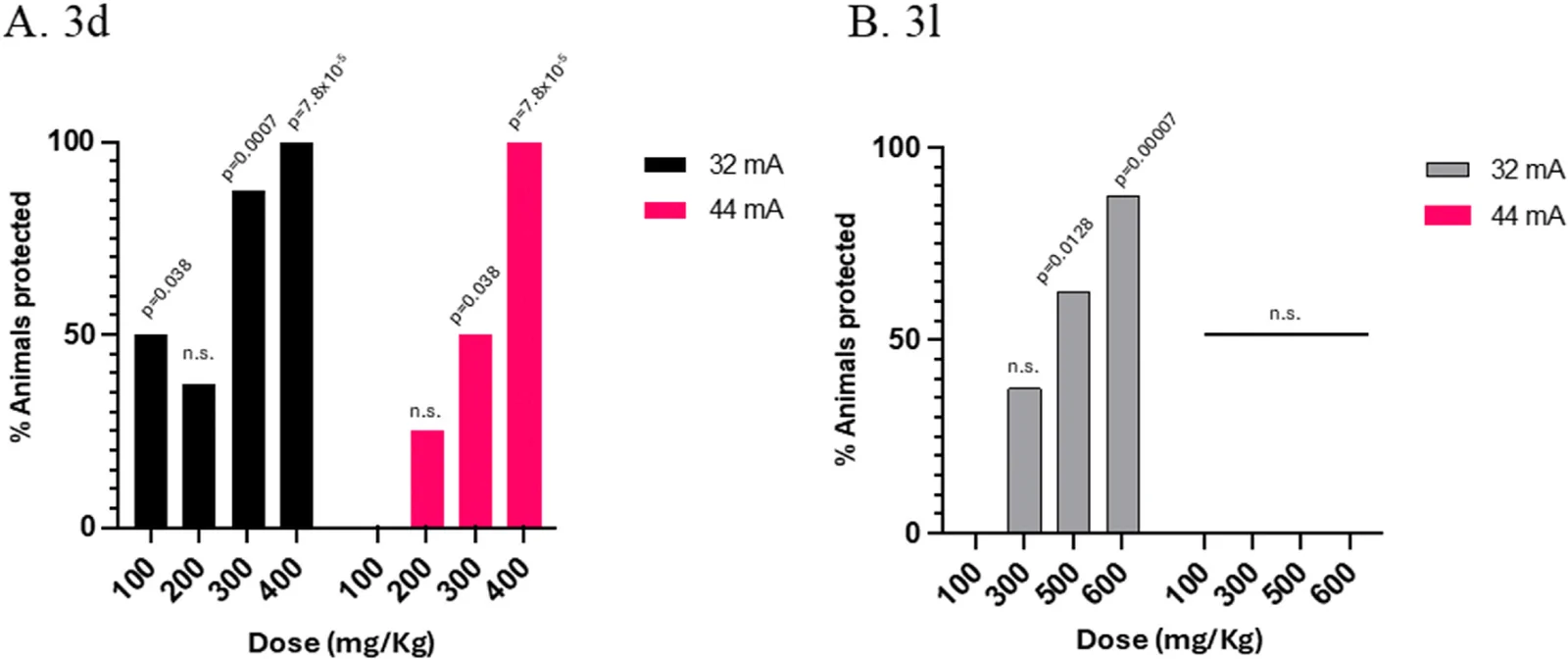
Comparative 6 Hz mouse seizure model bar graphs. Male CF-1 mice were treated with VK analog IP injections 25 min prior to the 6 Hz mouse seizure test. After electroshock, involuntary muscle movements in the jaw, forelimb, vibrissae, and tail were monitored as mouse seizure behavior. (A.) Compound 3d displayed a dose-dependent protection against convulsions in both 32 mA and 44 mA. (B.) Compound 3l also reduced seizures at 32 mA, but no longer worked at 44 mA. All concentrations in the range of 100–600 mg/kg. Each bar represents the percentage of animals protected after 6 Hz stimulation; n = 8 per condition. p values were calculated versus vehicle-treated seizure controls, and non-significant results are labeled as n.s.

Thirty-one compounds were funneled into three candidate VK analogs using three age groups in zebrafish. To validate the 21-dpf zebrafish results, the three compounds (3b, 3d, and 3l) that displayed efficacy at both 7 and 14-dpf were subsequently tested in adult mice. In Fig. 4, mice pretreated with 3d showed an effective dose-response in the 6 Hz seizure model at 32 mA and 44 mA. Protection increased in a dose-dependent manner. 3l demonstrated a protective dose response in mice at the lower 32 mA seizure threshold but no longer showed seizure protection at 44 mA. 3b was also tested at one concentration (300 mg/kg). This VK analog did not safeguard mice from seizures, and testing was discontinued.

### 3.2. CNS pharmacokinetic (PK) bioanalytical analysis in rodents

Two compounds (3d and 3l) were selected for the rodent PK bioanalytical analysis. A brain-to-plasma ratio above 0.2 is commonly used as supportive evidence of brain exposure (de Lange and Hammarlund-Udenaes, 2015; Fenyk-Melody et al., 2004; Hammarlund-Udenaes et al., 2008). Both 3d and 3l exceeded this threshold, with brain-to-plasma ratios of 2.64 and 0.853, respectively, indicating measurable CNS exposure consistent with their observed activity in zebrafish and mouse seizure assays (Table 1).

## 4. Discussion

### 4.1. Using zebrafish as an initial screen helps eliminate compounds less likely to penetrate the BBB prior to testing in mouse models

Using Baraban's PTZ-induced zebrafish seizure-like hyperlocomotor activity model, Rahn et al. revealed that novel VK analogs had potential as ASMs (Baraban et al., 2007; Josey et al., 2013; Rahn et al., 2014). These compounds could decrease hyperlocomotor activity in 7-dpf zebrafish and mouse seizure models. The research was focused on younger animal models to develop new ASMs for children (Rahn et al., 2014). Advanced VK analogs 3d and 3l produced approximately 50% reductions in PTZ-induced seizure-like hyperlocomotor activity in 7-dpf zebrafish (Li et al., 2020b). 7-dpf or younger fish are frequently used for drug testing. Furthermore, experiments utilizing 5-dpf or younger fish could be attributed to bypassing animal experimental regulations, where young zebrafish are not recognized as ‘animals’ until a certain age is passed (Cassar et al., 2020). We have tested VK analogs in older zebrafish beyond 10-dpf to determine whether they can diffuse through the fully developed BBB to reduce hyperlocomotor activity. 10-dpf zebrafish have been used to test FDA-approved CNS drugs crossing the BBB (Fleming et al., 2013). The available compounds that cross the BBB in humans were also able to pass in 10-dpf zebrafish, strengthening the argument that this age group does have a fully developed BBB (Fleming et al., 2013). Gabapentin decreased hyperlocomotor activity in adult zebrafish aged 3–4 and 5–6 months old (Banote et al., 2013; Pardridge, 2012). Phenytoin, Oxcarbazepine, Diazepam, and Rivastigmine successfully reduced PTZ-induced hyperlocomotor activity in 3–4 month fish; however, the fish received intraperitoneal injections of PTZ and the compounds (Kundap et al., 2017). There is some discussion as to whether PTZ works as effectively in fish older than 10-dpf, which may have resulted in the development of i.p.injection used in older fish (Kundap et al., 2017). However, we did not observe any issues with PTZ-induced seizure-like hyperlocomotor activity in 21-dpf zebrafish when PTZ was added to their swim media, as previously used with 7- and 14-dpf zebrafish. At 21-dpf, zebrafish given PTZ in their water were able to reach swim distances of 4000 mm, which closely matches the distance traveled for 7-dpf fish measured for the same amount of time. The VK analog results support previous ASMs passing the BBB in 7-dpf fish (Li et al., 2020b). Thirty-one VK analogs were screened across three zebrafish developmental stages to prioritize compounds for mammalian validation. Compounds 3b, 3d, and 3l were advanced to the 6 Hz mouse seizure model. Among these, 3d showed the strongest protection, while 3l was active at the lower seizure threshold but not at 44 mA, and 3b was inactive at the tested dose. PK analysis showed brain exposure for both 3d and 3l, with brain-to-plasma ratios of 2.64 and 0.853, respectively. The higher brain-to-plasma ratio of 3d is consistent with its stronger mouse efficacy, although further PK/PD studies are needed to define this relationship.

### 4.2. Later-stage zebrafish as an additional prioritization step for CNS-active compound screening

Compounds that can pass the BBB are often lipophilic and have a small molecular weight to allow transmembrane diffusion (Banks, 2009; Pardridge, 2012). According to Pajouhesh et al., (2005), in order to pass the BBB, pharmaceuticals’ topological polar surface area (tPSA) should be less than 90 Å, cLogP under 5, and molecular weight (MW) lower than 450 Da (Geldenhuys et al., 2015; Long et al., 2019; Pajouhesh and Lenz, 2005). Novel VK analogs are ideal naphthoquinone compounds, but high-throughput drug screening is needed to determine the most effective candidate (Rahn et al., 2014). Dietary VKs are fat-soluble compounds, but they cannot pass the BBB due to the lipophilic phenyl tail without some form of metabolic breakdown. VK tPSA is > 60 Å, with a molecular weight of 450 Da, and cLogP larger than 10. To expedite metabolic breakdown, newly generated VK analogs address the BBB penetration issue by altering configuration via lipophilic tail removal, the addition of two R groups, and a focus on the Y chemical group. The scaffolds remain attached to a lipophilic side chain, with a tPSA of <40 Å and a molecular weight of 400 Da. The cLogP is less than 5 (Josey et al., 2013; Li et al., 2020b).

Several factors may explain why compounds appeared to reduce PTZ-induced seizure-like hyperlocomotor activity more robustly in zebrafish than in mice. In zebrafish, compounds are delivered by immersion, and exposure is influenced by media concentration, compound solubility, stability, and uptake across external surfaces, including skin and gills (Cassar et al., 2020). In contrast, mice received defined systemic i.p. doses, where exposure depends on absorption, distribution, metabolism, clearance, and brain penetration. Thus, the zebrafish assay should be interpreted as an integrated phenotypic readout of compound potency, uptake, and CNS exposure.

Both 3d and 3l reduced PTZ-induced hyperlocomotor activity across zebrafish developmental stages. Compound 3d decreased locomotor activity by approximately 70%, 52%, and 45% in 7-, 14-, and 21-dpf zebrafish, respectively, whereas 3l reduced activity by approximately 85%, 87%, and 55% at the same stages (Fig. 3). In the mouse 6 Hz model, 3d showed stronger protection, whereas 3l was active at the lower 32 mA threshold but not at 44 mA. Compound 3b, which reduced PTZ-induced hyperlocomotor activity in 7- and 14-dpf zebrafish but was less active than 3d and 3l, did not protect mice at the tested 300 mg/kg dose. These findings suggest that later-stage zebrafish screening may help prioritize compounds for mammalian testing, while also highlighting the need for direct mammalian validation.

PK analysis further supported differences between 3d and 3l. Both compounds showed measurable brain exposure in mice, but 3d achieved a higher brain-to-plasma ratio than 3l at 30 min post-dose, with ratios of 2.64 and 0.853, respectively. This higher apparent CNS exposure for 3d is consistent with its stronger protection in the mouse 6 Hz model. However, because the PK analysis was limited to a single time point, additional time-course PK/PD studies will be required to define whether differences in brain entry rate, exposure duration, or target engagement explain the different efficacy profiles of 3d and 3l.

Regarding zebrafish BBB development, fluorescent tracers have demonstrated that gold nanoparticles accumulate inside the endothelial basement membrane in 3-dpf zebrafish. The markers were no longer located in this region after 5-dpf, suggesting the BBB's leakage and permeability drop off at this age (O'Brown et al., 2019). Another consideration in interpreting the zebrafish PTZ data is the potential role of BBB efflux transporters. In mammals, P-gp/ABCB1 and BCRP/ABCG2 are major ABC transporters at the BBB and can limit CNS exposure of many lipophilic small molecules. Zebrafish possess conserved BBB features, with functional maturation reported between approximately 3 and 10 dpf, and zebrafish homologs of mammalian efflux transporters have been identified. In particular, Abcb4 has been reported as the zebrafish P-gp homolog expressed at the BBB, and zebrafish ABCG2 homologs, including Abcg2a, may contribute to xenobiotic transport. Because many lipophilic compounds can be substrates for ABC efflux transporters, differential transporter activity across developmental stages may influence apparent efficacy in zebrafish. Thus, the larval PTZ assay should be interpreted as an integrated measure of compound potency, uptake, CNS exposure, and potential efflux liability. Future work will evaluate brain exposure and transporter interactions to better define the relationship between zebrafish efficacy and mammalian CNS pharmacokinetics.

**Fig. 5.**
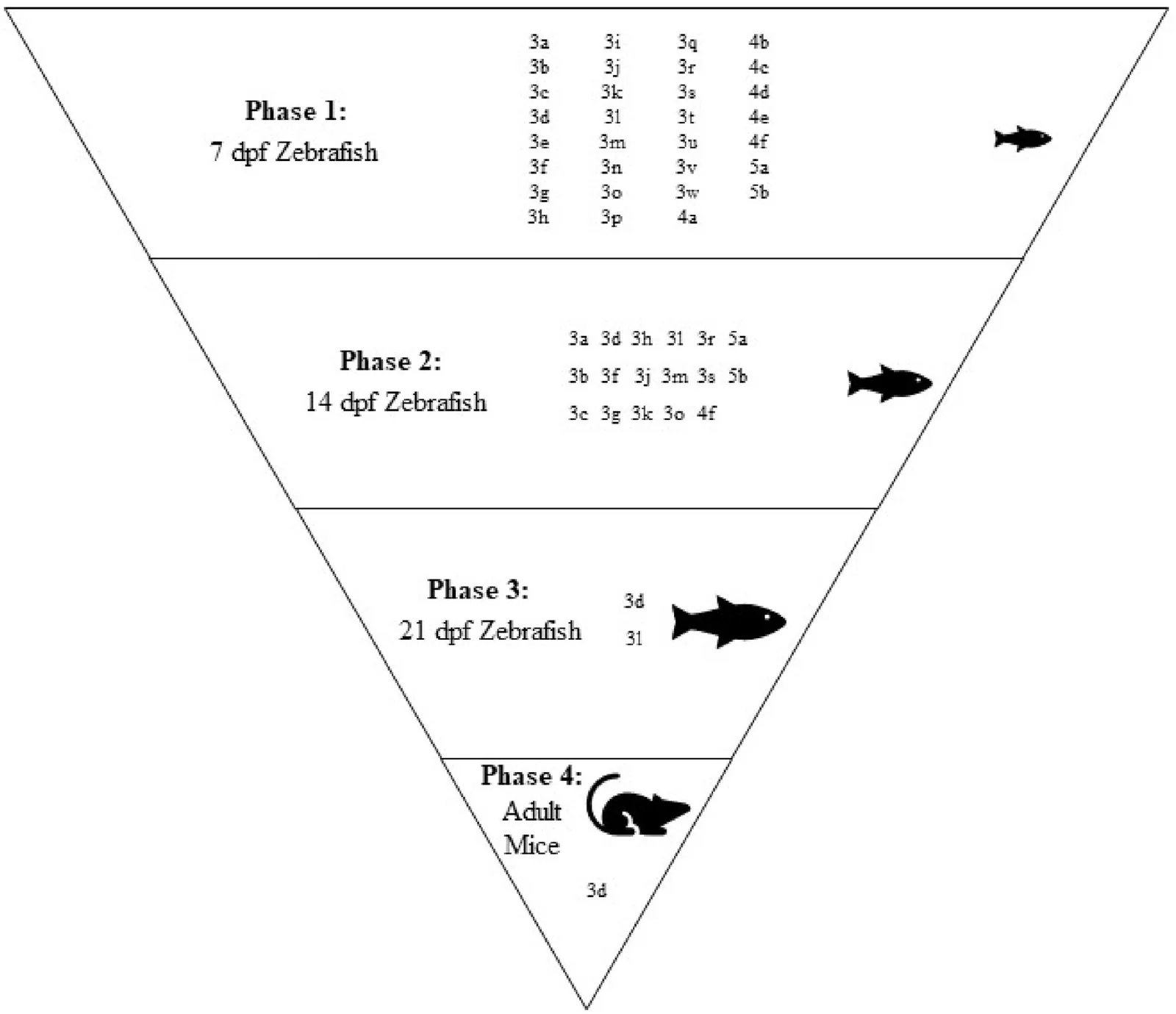
Proposed staged screening workflow using 7-, 14-, and 21-dpf zebrafish to prioritize compounds before mouse testing. Compounds were advanced based on the reduction of PTZ-induced seizure-like hyperlocomotor activity compared with PTZ control. Phase 1: Test compounds in 7-dpf zebrafish PTZ-induced hyperlocomotor activity model. Phase 2: Test compounds showing reduction in PTZ-induced hyperlocomotor activity with p < 0.05 values in 14-dpf zebrafish. Phase 3: Test compounds that reduce PTZ-induced hyperlocomotor activity in 14-dpf zebrafish with p < 0.05 values in 21-dpf zebrafish. Phase 4: Test compounds active in 21-dpf zebrafish in the 6 Hz mouse model.

Yet with this information, we were unable to conclusively determine whether the BBB is fully formed in 7- and 14-dpf larvae. These findings support the use of 21-dpf zebrafish as an additional prioritization step before rodent testing. In the future, it may save more time by testing compounds only in 21-dpf zebrafish; however, more effort and care are needed to rear sufficient numbers of fish to this age for experiments. Thus, we recommend that to improve prioritization of CNS-active compounds before mammalian testing, and to potentially save time and cost of using mammalian models, researchers should consider testing the drugs in 4 different phases: Phase 1 in 7-dpf, phase 2 in 14-dpf, and phase 3 in 21-dpf zebrafish, preemptively before testing in phase 4 in mice (Fig. 5).

### 4.3. Limitations of the studies

A limitation of this study is that zebrafish seizure-like activity was assessed using locomotor distance rather than electrophysiological recordings. PTZ-induced hyperlocomotion is a widely used behavioral endpoint in zebrafish seizure screening, but it cannot fully distinguish suppression of epileptiform activity from nonspecific effects such as sedation, reduced motor activity, toxicity, or altered compound uptake. In addition, zebrafish differ from mammals in neuroanatomical and circuit organization, including the absence of mammalian cortical circuitry. Therefore, the zebrafish data should be interpreted as a phenotypic prioritization screen rather than definitive proof of antiseizure efficacy. The subsequent 6 Hz mouse model was used as an orthogonal mammalian validation step, and future studies will include zebrafish local field potential or EEG recordings to directly assess epileptiform activity.

Another limitation of this study is the reliance on PTZ-induced seizure-like behavior. PTZ is a GABA_A receptor antagonist and therefore may preferentially identify compounds active against seizures driven by reduced inhibitory signaling. Compounds inactive in PTZ models may still have antiseizure activity through other mechanisms, including sodium-channel modulation. However, zebrafish PTZ assays have also shown responsiveness to several non-GABAergic ASMs (Milder et al., 2022), indicating that the model is useful as a phenotypic screen but not a universal predictor of ASM activity. Future studies should evaluate selected compounds in additional zebrafish seizure paradigms and include electrophysiological confirmation.

The in vivo formulation is the use of DMSO as a co-solvent. The compounds are poorly water-soluble, and 5% DMSO was required to maintain a useable dosing formulation. Although this concentration is commonly used in short-term rodent studies and has been reported to be tolerated in mice under repeated i.p. dosing conditions, DMSO is biologically active and can alter membrane properties and permeability. Therefore, potential effects of DMSO on tissue distribution or apparent exposure cannot be excluded. To minimize this confounder, the same DMSO-containing vehicle was used in the control and treatment groups. Future formulation optimization will focus on reducing or eliminating DMSO through alternative vehicles or solubilizing excipients. In zebrafish assays, DMSO concentration was kept consistent across vehicle and treatment groups. DMSO concentrations up to 1% have been reported to be tolerated in zebrafish embryo developmental toxicity assays without increased lethality or malformations (Hoyberghs et al., 2021); however, DMSO can alter larval locomotor behavior at sublethal concentrations, supporting the use of matched vehicle controls in behavioral assays (Christou et al., 2020).

Although the staged zebrafish approach should not be interpreted as direct proof of improved translational predictability, it provides a practical and scalable strategy for prioritizing CNS-active compounds before mammalian testing. Zebrafish immersion exposure differs from systemic dosing in mammals and is influenced by compound solubility, media stability, external uptake, developmental stage, and differences between nominal media concentration and internal brain exposure. Even with these limitations, later-stage zebrafish screening can serve as a useful intermediate filter to guide compound selection for subsequent mammalian efficacy, PK/PD, and electrophysiological studies.

## Supplementary Material

Supplementary Table 1

## Figures and Tables

**Table 1:** Plasma and brain concentrations and brain-to-plasma ratios in mice. Compounds 3d and 3l were I.V. injected into male CF-1 mice at doses of 2–5 mg/kg for 30 min 3d and 3l displayed measurable brain-to-plasma concentration ratios, supporting CNS exposure. Data analysis for concentration vs. time was performed using individual animals’ means, standard deviations, and coefficients of variation.

Compound	Matrix	Time (hr)	Homogenate Mean Concentration (ng/mL or ng/ g)	Brain-to-plasma ratio

3d	Plasma	0.5	1257 (±135) CV 10.7%	N/A
3d	Brain	0.5	3320 (±208) CV 6.27%	**2.64**

3l	Plasma	0.5	586 (±18.4) CV 3.14%	N/A
3l	Brain	0.5	500 (±45.2) CV 9.05%	**0.853**

## Appendix A. Supplementary data

Supplementary data to this article can be found online at https://doi.org/10.1016/j.neuropharm.2026.111085.
