# Supplementary material for: A staged zebrafish–mouse screening strategy for antiseizure compound prioritization
Source: Neuropharmacology. Author manuscript; Available in PMC 2026 Sep 9. (PMC13557030; doi:10.1016/j.neuropharm.2026.111085)
Supplement: Supplementary file 1 [file NIHMS2197975-supplement-Supplementary_Table_1.docx]

Supplemental Table 1: Thirty-one VK analogs with compound structures initially tested on 7 dpf PTZ-treated zebrafish.

| **Compound Numbers** | **Structure** | **Compound Numbers** | **Structure** | **Compound Numbers** | **Structure** |
| --- | --- | --- | --- | --- | --- |
| 3a |  | 3l |  | 3w |  |
| 3b |  | 3m |  | 4a |  |
| 3c |  | 3n |  | 4b |  |
| 3d |  | 3o |  | 4c |  |
| 3e |  | 3p |  | 4d |  |
| 3f |  | 3q |  | 4e |  |
| 3g |  | 3r |  | 4f |  |
| 3h |  | 3s |  | 5a |  |
| 3i |  | 3t |  | 5b |  |
| 3j |  | 3u |  | VK1 |  |
| 3k |  | 3v |  | VK2 (MK4 n=4) |  |
